# MSI2 is required for maintaining activated myelodysplastic syndrome stem cells

**DOI:** 10.1038/ncomms10739

**Published:** 2016-02-22

**Authors:** James Taggart, Tzu-Chieh Ho, Elianna Amin, Haiming Xu, Trevor S. Barlowe, Alexendar R. Perez, Benjamin H. Durham, Patrick Tivnan, Rachel Okabe, Arthur Chow, Ly Vu, Sun Mi Park, Camila Prieto, Christopher Famulare, Minal Patel, Christopher J. Lengner, Amit Verma, Gail Roboz, Monica Guzman, Virginia M. Klimek, Omar Abdel-Wahab, Christina Leslie, Stephen D. Nimer, Michael G. Kharas

**Affiliations:** 1 Molecular Pharmacology and Center for Cell Engineering, Center for Stem Cell Biology, Center for Experimental Therapeutics, Memorial Sloan Kettering Cancer Center, New York, New York 10065, USA; 2 Memorial Sloan Kettering Cancer Center, Cancer Biology Program, New York, New York 10065, USA; 3 Computational Biology Program Memorial Sloan Kettering Cancer Center, Memorial Sloan Kettering Institute, New York, New York 10065, USA; 4 Memorial Sloan Kettering Cancer Center, Human Oncology and Pathogenesis Program, New York, New York 10065, USA; 5 Memorial Sloan Kettering Cancer Center, Department of Medicine, Leukemia Service, New York, New York 10065, USA; 6 Department of Animal Biology, Department of Cell and Developmental Biology and Institute for Regenerative Medicine, Schools of Veterinary Medicine and Medicine, University of Pennsylvania, Philadelphia, Pennsylvania 19104, USA; 7 Montefiore Medical Center, Albert Einstein College of Medicine, Bronx, New York 10461, USA; 8 Joan and Sanford I. Weill Department of Medicine, Weill Cornell Medical College, New York, New York 10065, USA; 9 Division of Hematology and Medical Oncology, Department of Medicine and Pharmacology, Weill Cornell Medical College, Cornell University, New York, New York 10065, USA; 10 Sylvester Comprehensive Cancer Center, Department of Medicine, Miller School of Medicine, University of Miami, Miami, Florida 33136, USA

## Abstract

Myelodysplastic syndromes (MDS) are driven by complex genetic and epigenetic alterations. The MSI2 RNA-binding protein has been demonstrated to have a role in acute myeloid leukaemia and stem cell function, but its role in MDS is unknown. Here, we demonstrate that elevated MSI2 expression correlates with poor survival in MDS. Conditional deletion of Msi2 in a mouse model of MDS results in a rapid loss of MDS haematopoietic stem and progenitor cells (HSPCs) and reverses the clinical features of MDS. Inversely, inducible overexpression of MSI2 drives myeloid disease progression. The MDS HSPCs remain dependent on MSI2 expression after disease initiation. Furthermore, MSI2 expression expands and maintains a more activated (G1) MDS HSPC. Gene expression profiling of HSPCs from the MSI2 MDS mice identifies a signature that correlates with poor survival in MDS patients. Overall, we identify a role for MSI2 in MDS representing a therapeutic target in this disease.

## Abstract (web summary)

Several studies have recently demonstrated the role of the MSI2 RNA binding protein in normal and malignant haematopoietc stem cells. In this study, the authors show that MSI2 is required for maintaining myelodysplastic syndrome stem cells in mice and that MSI2 expression predicts poor prognosis in patients affected by this disease.

## Graphical abstract

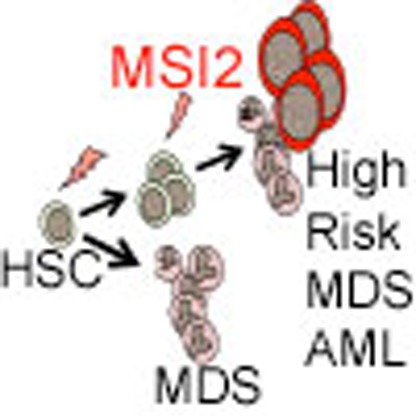

The majority of haematological disorders involving the myeloid lineage are thought to be of stem cell origin, including myeloproliferative diseases, myelodysplastic syndromes, acute myeloid leukaemia and acquired or heritable bone marrow failure syndromes^1^,^2^,^3^. In each instance, dysregulation of normal stem cell function is thought to contribute to the disease phenotype. Moreover, stem cell characteristics are modulated by a variety of developmental pathways and regulators. Recent studies of MSI2 in normal and malignant hematopoietic stem cell (HSC) biology suggested that MSI2 might play a role in myelodysplastic syndromes (MDS)^4^,^5^,^6^,^7^,^8^,^9^,^10^,^11^. It was previously reported that *MSI2* expression in MDS was reduced in patients with low-risk and high-risk MDS compared with normal CD34 cells^7^. However, in this study there was a subset of MDS patients with excess blasts with increased *MSI2* (ref. 7). The functional importance of MSI2 in MDS therefore remains unclear. We examine previously published expression data sets and patient samples to find that MSI2 is increased in high-risk MDS patients. Additionally, we utilize MSI2 loss and gain of function approaches in the context of a mouse model of MDS and find that MSI2 is required for MDS.

## Results

### Elevated MSI2 expression predicts poor survival in MDS

**Figure 1:**
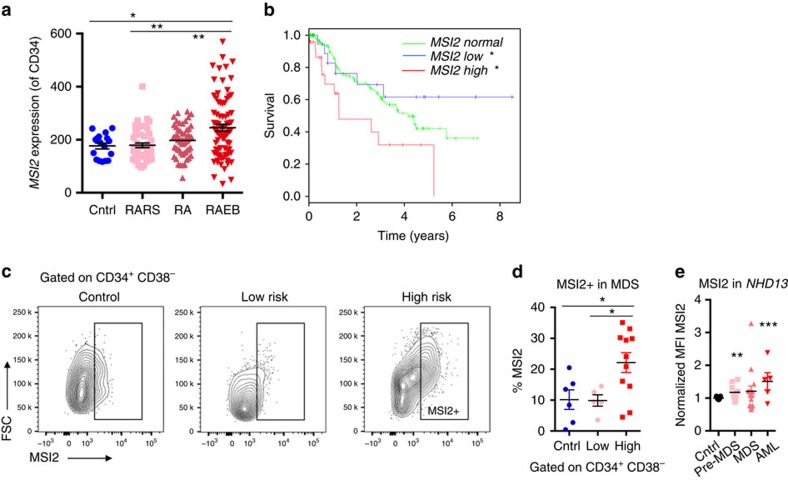
Elevated MSI2 expression predicts poor survival in MDS. (**a**) Microarray expression data (CD34^+^ population) from normal elderly individuals (CD34+; *n*=17) and MDS (*n*=183), RARS, RA, RAEB, *P<0.05, **P<0.01, ***P<0.001 Student's *t*-test mean±s.e.m^12^. (**b**) Overall survival in MDS patients stratified by *MSI2* expression (as high (*Z*-score>1), low (*Z*-score<−1), or normal (−1≤*Z*-score≤1) log-rank test. (**c**). Representative flow cytometric analysis of independent patient cohort of primary patients samples gated on CD34^+^CD38^−^ and stained for intracellular MSI2, age-matched elderly individuals (*n*=6), low risk (RA or RARS; *n*=5) and high risk (RAEB-1, RAEB-2 *n*=10) (**d**). MSI2 positivity summarized from gating of patients in **c**. (**e**) MSI2 intracellular levels in the *NHD13* MDS/AML animal model. Cells are initially gated on MSI2-positive cells (Supplementary Fig. 1 for gating) and median fluorescence intensity (MFI) is normalized to the control (C57BL6 mice), *n*=19. Pre-MDS represents the analysis preformed in *NHD13 *primary or transplanted mice within 1–2 months of birth and before MDS onset (*n*=9), the MDS mice that are older than 2 months or primary transplanted and have low WBC, (*n*=16) and AML samples are from *NHD13 *mice that have transformed to AML (*n*=5), **d** and **e** *P<0.05, **P<0.01, ***P<0.001 Student's *t*-test horizontal line is the mean±s.e.m.

In our examination of a previously published expression data set, we found that *MSI2* expression was increased in CD34^+^ population in high-risk MDS patients (refractroy anemia with excess blasts; RAEB) compared with healthy individuals that were not age matched or Low-Risk MDS (Refractory Anemia; RA or refractory anemia with ringed sideroblasts; RARS), Fig. 1a)^12^. Elevated MSI2 levels correlated with a poor clinical survival (Fig. 1b and Supplementary Fig. 1a). In line with the microarray data, high-risk MDS patients had increased intracellular MSI2 in their CD34^+^CD38^−^ cells compared with low-risk MDS patients and healthy individuals (Fig. 1c,d). Altogether, the MDS patient data suggests that the level of *MSI2* expression correlates with disease subtype and clinical outcome. In contrast to the acute myelogenous leukemia (AML) patient data, where elevated *MSI2* expression correlates with FLT3-ITD/NPM1 mutations^5^,^8^,^9^,^11^, MDS patients do not typically harbour these mutations. Due to the low number of patients with recurrent mutations in this study, we are unable to correlate MSI2 levels with individual mutations (Supplementary Table 1).

### Msi2 is required for MDS

To test if Msi2 could be functionally important in MDS, we utilized a murine model of MDS. The *NUP98-HOXD13* transgenic model (*NHD13*) recapitulates many of the salient features of MDS, including neutropenia, lymphopenia and hypercellular or normocellular bone marrow at 4–7 months^13^,^14^,^15^,^16^. Also, 12–17% of the marrow contains dysplastic erythroid, myeloid and rare megakaryocytic cell types^13^. Similar to patients with MDS, a significant cohort of the primary mice can progress and develop an aggressive AML. However, if the bone marrow of *NHD13* mice is transplanted, the recipient animals succumb to a fully penetrant but non-lethal form of MDS that rarely progresses to AML (ref. 15). Although the *NHD13* transplanted bone marrow cells engraft poorly, they still retain the clinical features of MDS (∼10–20% peripheral blood chimerism)^15^. Utilizing intracellular staining for MSI2, we found a significant albeit modest increase in MSI2 levels in the bone marrow of 44% of NHD13 pre-MDS, 50% of MDS, and 80% of AML animals (Fig. 1e and Supplementary Fig. 1b). The significant increase in MSI2 was also observed within the sorted progenitors from pre-MDS animals (Supplementary Fig. 1c,d).

**Figure 2:**
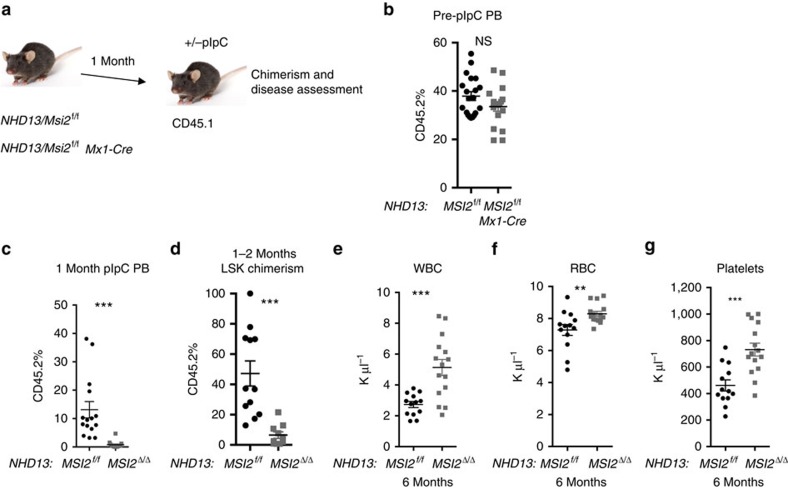
*Msi2* is required to maintain MDS. (**a**) Transplant scheme for Msi2 conditional knockout in *NHD13* MDS model. (**b**) Peripheral blood chimerism 4 weeks posttransplantation as described in **a**, (*NHD13/Msi2*^f/f^ *n*=20 and *NHD13*/*MSI2*^*f/f*^ *Mx1-Cre; n*=18). (**c**) Peripheral blood chimerism 8-weeks posttransplantation (4-weeks post-pIpC treatment; *NHD13/Msi*^f/f^ *n*=15 and *NHD13/Msi2*^*Δ*/*Δ*^*Mx1-Cre; n*=14). (**d**) Chimerism within the haematopoietic stem and progenitor cell compartment from bone marrow aspirates performed 8-weeks posttransplantation, gated on Lin-Sca1^+^Kit^+^ cells, (*NHD13/Msi2*^f/f^ *n*=12 and *NHD13/Msi2*^*Δ*/*Δ*^*Mx1-Cre; n*=10). (**e**) WBC. (**f**) Red blood cells (RBC). (**g**) Platelets at 6 months posttransplant, **f**–**h**, (analysed at 6 months post-pIpC, *NHD13/Msi2*^f/f^ *n*=13 and *NHD13/Msi2*^*Δ*/*Δ*^*Mx1-Cre; n*=15). Data in **b**–**g** are represented two independent transplants *P<0.05, **P<0.01, ***P<0.001, Student's *t*-test horizontal line is the mean±s.e.m.

In agreement with MDS patient data, we observed an increase in the expression of MSI2 in the *NHD13* mice during disease progression. These data suggested that altering MSI2 levels in the *NHD13* model could alter the disease fate. To test this hypothesis, *Msi2* conditional knockout were crossed with the *NHD13* mice and then transplanted into congenic recipients (Fig. 2a,b). The chimerism in the peripheral blood and at the level of the haematopoietic stem and progenitor cell (HSPC) was significantly reduced one month after pIpC-mediated deletion (Fig. 2c,d). *Msi2* deletion resulted in the loss of the *NHD13*-expressing cells and a reversal of MDS-like disease that included an increase in white blood cell (WBC) counts, red blood cells and platelets (Fig. 2e–g). When the mice were analyzed 14 months after transplantation there was a trend towards reduced spleen weight, normalized WBC counts and significantly reduced chimerism in the HSPCs and in progenitors (Supplementary Fig. 2a–c). Despite the fact that some of the mice had detectable donor chimerism, the donor myeloid cells were mainly absent and the few remaining donor cells retained MSI2 expression indicating that the deleted cells were selected against (Supplementary Fig. 2d,e). Nevertheless, these mice did not have detectable dysplastic cells or leukaemia in their bone marrow (Supplementary Fig. 2f).

### MSI2 overexpression in MDS drives transformation

**Figure 3:**
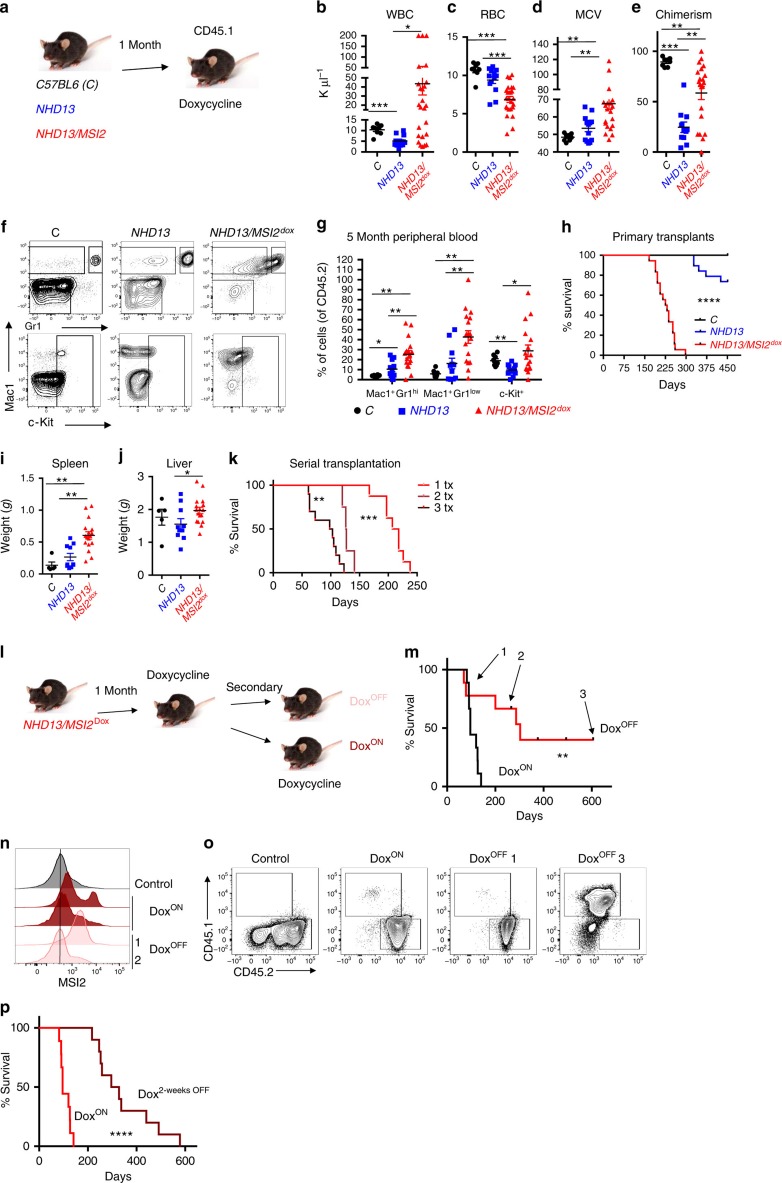
Sustained MSI2 overexpression transforms MDS to a lethal AML. (**a**) Experimental scheme for MSI2 overexpression in the *NHD13* transplant model. (**b**) Peripheral blood analysis 5 months posttransplantation, including WBC. (**c**) Red blood cell count (RBC). (**d**) Mean corpuscular volume (MCV). Data in **b**–**d**, (C; *n*=8, *NHD13*; *n*=13, *NHD13/MSI2*; *n*=25). (**e**) Chimerism (CD45.2) in the peripheral blood 5 months posttransplantation, (C; *n*=8, *NHD13*; *n*=11, *NHD13/MSI2*; *n*=21). (**f**) Representative Mac1, Gr1, and c-Kit staining of peripheral blood from the bone marrow. Mice were analysed 5 months posttransplant and gated on CD45.2^+^ cells. (**g**) Immunophenotyping of peripheral blood 5 months posttransplant. Samples gated as in **e**, data in **f** and **g**, (C; *n*=8, *NHD13*; *n*=11, *NHD13/MSI2*; *n*=17). (**h**) Survival curves of *NHD13* with MSI2 overexpression combined from two independent transplants mice (C; *n*=8, *NHD13*; *n*=15, *NHD13/MSI2*^Dox^; *n*=18). (**i**) Spleen and (**j**) liver weights from healthy mice analysed at the end point or moribund mice, (C; *n*=5, *NHD13*; *n*=10, *NHD13/MSI2*; *n*=17). (**k**) Survival analysis of serially transplanted myeloid disease in the *NHD13/MSI2*^Dox^ mice (primary donor; *n*=8, secondary; *n*=4 and tertiary transplants; *n*=10; transplanted from two independent donors). (**l**) Experimental scheme for testing MSI2 dependence of *NHD13*/*MSI2* AML. The bone marrow from moribund primary *NHD13*/*MSI2* transplanted animals was secondarily transplanted into congenic mice and treated accordingly. (**m**) Survival analysis for doxycycline on/off secondary transplants, (Dox^ON^; *n*=8 and Dox^OFF^; *n*=8 from two independent donors and transplants), Arrows 1, 2 and 3 indicate three representative mice that are described in **n** and **o**. (**n**) Intracellular MSI2 staining by flow cytometry in fixed bone marrows of representative moribund mice described in **m**. (**o**) Representative CD45.1/2 staining of mice at different points in the survival curve from **m**. (**p**) Survival analysis of doxycycline treated *NHD13*/*MSI2* secondary transplants relative to transplants whose recipients had a 2-week delay in doxycycline treatment posttransplant, Dox^ON^; *n*=9 and Dox^OFF^; *n*=10 from two independent transplants). Data in **b**–**k**, are represented two independent transplants *P<0.05, **P<0.01, ***P<0.001, **b**–**e**, **g**,**i** and **j** are Student's *t*-test and horizontal line is the mean±s.e.m and **h**,**k**,**m** and **p**, *P* values assessed by log-rank.

We next assessed if forced MSI2 expression could alter the disease course using the same model of MDS. Of note, MSI2 overexpression by itself does not result in leukaemic transformation^5^. We utilized our previously described inducible MSI2 overexpressing mouse model that can be controlled with doxycycline (*KH2-Col1A1-tet-on-MSI2/ROSA26*-rTTA) and crossed them with the *NHD13* mice^5^. Control (C57BL/6), *NHD13* or *NHD13/MSI2* bone marrow was transplanted into congenic mice and then allowed to engraft before MSI2 was induced (Fig. 3a). After 5 months the *NHD13/MSI2* overexpression mice started to succumb to lethal myeloid diseases while the *NHD13* mice had symptoms of a mild MDS. The *NHD13/MSI2* mice had reduced WBC (5/25) or elevated WBC counts, reduced red blood cell counts, increased mean corpuscular volume and increased chimerism in the blood at 5 months post-transplantation compared with the control and the *NHD13* mice (Fig. 3b–e). We observed increased immature myeloid cells in the peripheral blood, and all of the MSI2 overexpressing *NHD13* mice eventually succumbed to various lethal myeloid diseases including MPN/MDS or an AML/MDS with a median latency of 228 days (Fig. 3f–h and Supplementary Fig. 3a–g). At end point, we found that the *NHD13/MSI2* mice had a more severe disease burden based on increased spleen and liver weights compared with the control and *NHD13* mice (Fig. 3i,j). The *NHD13* mice showed signs of a MPN/MDS or MDS disease, but only 3 out 15 mice died of a characterized myeloid disease (AML/MDS *n*=2, MDS *n*=1, and one mouse was found dead and another died of a non-myeloid disease; Fig. 3i,j). Serial transplantation of the *NHD13/MSI2* demonstrated reduced latency further supporting the idea that MSI2 overexpression resulted in a clonal myeloid disease (Fig. 3k and Supplementary Fig. 3h). We secondarily transplanted the *NHD13* AMLs and then compared the disease burden to the secondary transplants from the *NHD13/MSI2* mice. Despite the fact that both groups had myeloid disease, the *NHD13/MSI2* group retained their more aggressive phenotype compared with the *NHD13* AMLs indicated by the increased spleen and liver weights (Supplementary Fig. 3i,j).

### MSI2 maintains activated MDS stem and progenitor cells

We then determined if MSI2 overexpression was required to maintain the disease. Thus, we transplanted *NHD13/MSI2* overexpressing mice into secondary recipients with or without doxycycline feed. Mice that were maintained on doxycycline and expressed MSI2 rapidly formed a lethal myeloid disease, while the majority of mice that were no longer being induced survived significantly longer (median, 96 versus 303 days; Fig. 3l,m and Supplementary Fig. 3k–t). Moreover, in mice that were no longer induced and died at the same time as the mice in the induced group, we were still able to detect high levels of intracellular MSI2 suggesting selection for constitutive activation *in vivo* (Fig. 3n). Similarly, a mouse that died later also demonstrated leaky MSI2 expression albeit at lower levels compared with the mouse that died earlier. Overall, in all the mice that died of leukaemia, MSI2-positive cells were detectable. However, mice killed at the experimental end point that remained disease free, we found that the chimerism was either low or undetectable (Fig. 3o). To further examine if transient withdrawal of MSI2 expression could also delay the leukaemia, we transplanted *NHD13/MSI2* cells and waited 2 weeks to induce MSI2 expression (Fig. 3p). We observed a delay in the myeloid leukaemia (312 days compared with 96 days) in the control, and these leukaemias relapsed with MSI2 positivity, providing evidence that MSI2 overexpression must be sustained to maintain disease.

**Figure 4:**
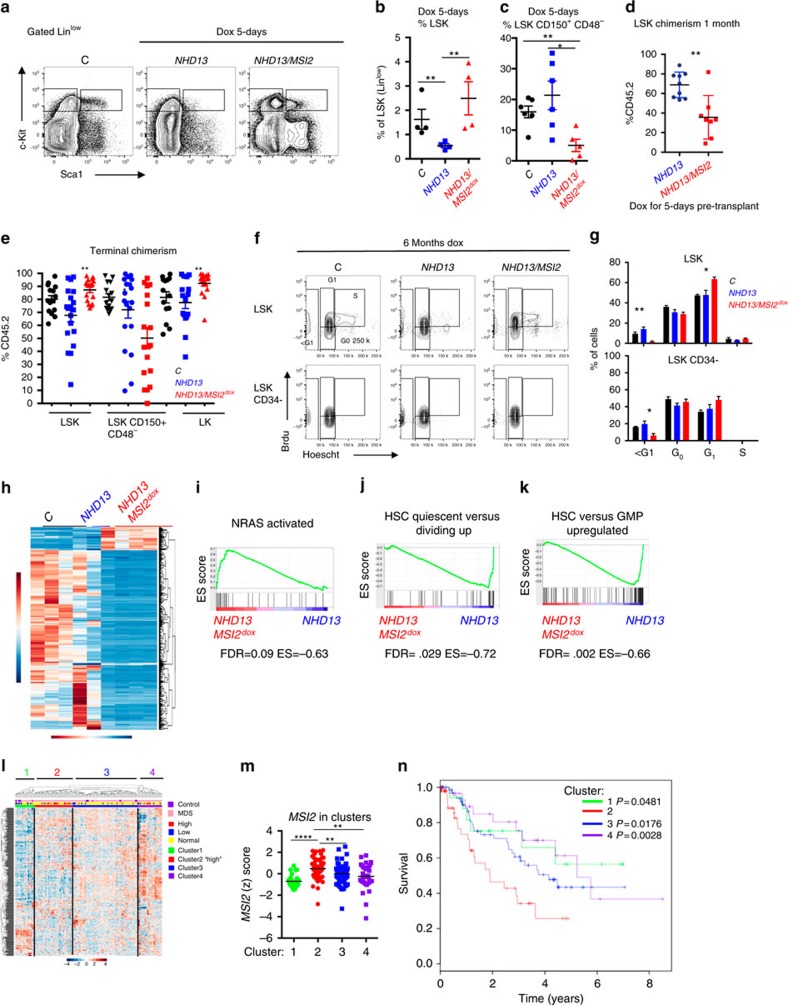
MSI2 overexpression leads to haematopoietic progenitor stem cell expansion. (**a**) Representative flow cytometry of primary animals treated with doxycycline (Dox) for 5 days and live and Lin^−^ gated. (**b**) Percentage of LSK population among the Lin^low^ compartment from primary mice (C; *NHD13* and *NHD13/MSI2*^Dox^; *n*=6, *n*=6 and *n*=5 from five independent experiments). (**c**) Percentage of the HSC (LSK^+^CD150^+^CD48^−^) compartment gated from the LSK^+^ at 1 month transplantation after 5 day dox administration in the primary animals, data representative (C; *NHD13* and *NHD13/MSI2*^Dox^ same as **b**). (**d**) Chimerism (CD45.2) in the LSK compartment at 1 month transplantation after 5 day dox administration in the primary animals, data representative of two independent transplants (*NHD13*; *n*=9, *NHD13/MSI2*^Dox^; *n*=8), (**e**) Terminal chimerism from transplants in Fig. 3 in the gated populations, (C, *NHD13*, *NHD13/MSI2*^Dox^; *n*=11-16 combined from two independent transplants). (**f**) Representative flow cytometric plots from transplanted mice analysed (combined experiments from 3 and 7 months posttransplant) that were injected with Brdu 24 h and then sorted for LSK cells and gated accordingly. (**g**) Data represented in **f**, (*n*=3 for each group combined from two independent experiments). (**h**) RNA-sequencing of LSK sorted cells from primary transplanted animals (4 months posttransplantation and 3 months post MSI2 induction) before disease initiation underwent unsupervised clustering of the differentially expressed genes with human homologues, (C; *n*=3, *NHD13*; *n*=2, *NHD13/MSI2*^Dox^; *n*=4). (**i**–**k**) Gene set enrichment analysis (GSEA) from ranked list of *NHD13/MSI2*^Dox^/*NHD13* versus Control. (**l**) Unsupervised clustering of the mouse *NHD13* signature overlapped with MDS patients. (**m**) *MSI2* expression (*Z*-score) separated based on clustering in **l**. (**n**) Survival of MDS patients based on clusters from the *NHD13/MSI2* signature (**l**,**m**). Data in **b**–**e**,**g** and **m** *P<0.05, **P<0.01, ***P<0.001, are Student's *t*-test and the horizontal line is the mean±s.e.m and **n**, *P* values are displayed and calculated with log-rank.

*NHD13* haematopoietic cells have a block in their differentiation at the HSC to multipotent progenitors (MPP) stage and have dramatically reduced numbers of HSPCs (ref. 16; Fig. 4a,b). MSI2 induction for 5 days resulted in an increase in the percentage of phenotypic LSKs (Lin-Sca1^+^Kit^+^ cells) and a decrease in the phenotypic HSCs, but no difference in the frequency of the myeloid or erythroid progenitors (Fig. 4a–c and Supplementary Fig. 4a). Interestingly, if the 5-day-induced *NHD13/MSI2* cells were then transplanted in the absence of doxycycline to turn off MSI2, we observed reduced chimerism at 1 month (Fig. 4d). Alternatively, when non-induced bone marrow was engrafted and then activated for MSI2, the LSK compartment was expanded at 1 month (Supplementary Fig. 4b), and in the diseased *NHD13/MSI2* mice LSKs and myeloid progenitors (granulocyte-monocyte progenitor (GMP) and common myeloid progenitor (CMP)) were increased compared with the *NHD13* mice (Fig. 4e and Supplementary Fig. 4c). Similarly to the 5-day induction the chimerism of the phenotypic HSCs (LSK^+^CD150^+^;CD48) was reduced in the diseased *NHD13/MSI2* compared with the *NHD13* and the control animals. We then profiled the cell cycle status of the HSPCs using Brdu incorporation and Hoechst staining and found reduced cell death (sub-G1) and increased percentage of cells in G1, which suggests the accumulation of more activated HSPCs (Fig. 4f,g). Taken together with the previous data, MSI2 expression maintains a more aggressive myeloid disease and a more activated HSPC.

To further characterize how MSI2 alters the *NHD13* MDS programme in the dysregulated stem cell compartment, we performed transcriptome profiling in the HSPCs (LSK) from transplanted mice after 3 months of doxycycline administration and before the mice demonstrate any disease phenotype. To elucidate the *NHD13/MSI2* expression programme, we utilized a generalized linear model that identified 891 significant genes (q-value <0.01, generalized linear model), of which 137 genes were upregulated (log2 fold change >0) and 754 genes were downregulated (log2 fold change ≤0). We then matched the gene signature to human homologues (690 genes; Supplementary Data 1) and created a heatmap after unsupervised hierarchical clustering, which separated the samples into their respective groups (Fig. 4h).

To functionally annotate our RNA-sequencing, we performed gene set enrichment analysis^17^ on all curated gene sets in the molecular signatures database (http://www.broadinstitute.org/msigdb; 3,256 gene sets) combined with an additional set of relevant gene sets (92 gene sets from our experimentally derived or published haematopoietic self-renewal and differentiation signatures^4^,^17^; rank list; Supplementary Data 2). We found 14 gene sets that were enriched for genes that were upregulated and 29 gene sets enriched for downregulated genes (Supplementary Tables 2 and 3). The top ranked gene sets included enrichment in an *NRAS* activated signature^18^, a reduced quiescent phenotype^19^ and a more progenitor-like cell (Fig. 4i–k). Taken together with our phenotypic analysis of the HSPC compartment, MSI2 induction increases the cells that are in G1, switching them to a less quiescent and more progenitor-like gene expression signature.

To determine if the MSI2 signature from the murine model of MDS corresponds to patients with MDS, we overlapped the *NHD13/MSI2* RNA-seq (690 genes) with microarray data from control (*n*=17) and MDS patients (*n*=183) (refs 12, 20). After unsupervised clustering of the human microarray data, we obtained four distinct clusters (Fig. 4l). Patients with elevated MSI2 expression were mainly found in Cluster-2, which predicted a poor survival compared with the other clusters (Fig. 4m,n). Our study demonstrates an important functional role of MSI2 in MDS (Supplementary Fig. 4d).

## Discussion

In summary, we found that elevated MSI2 expression predicts poor prognosis in MDS and is required for maintaining the diseased MDS stem cell. Cooperativity with *NHD13* has been associated with various factors including FLT3, MEIS1, P16 and TP53 (refs 16, 21, 22, 23). MSI2 overexpression can act as a cooperating oncogene and drive transformation, accelerate leukaemia and increase disease burden in the context of a MDS mouse model. Additionally, we found reduced apoptosis and a more activated stem cell, suggesting that the altered HSPC may contribute to disease progression. Gene expression profiling of HSPCs from the *NHD13/MSI2* mice generated a signature that overlapped with human MDS and could predict patient outcome. Our lab previously found that MSI2 directly binds to the mRNA of mixed-lineage leukaemia target genes including *Hoxa9*, *Myc* and *Ikzf2*, and regulates the translation of these targets in a mixed-lineage leukaemia-AF9 leukaemia model^9^. Additionally, a recent report showed that Msi2 may regulate the development and propagation of AML through Tetraspanin 3 (refs 24). Future studies will determine how MSI2 alter stem cells in MDS or whether it uses similar mechanisms as in AML.

Several studies have demonstrated that *Msi2* is required for HSPC engraftment^4^,^10^. It is unclear if in the context of suppressed hematopoiesis where few normal HSCs remain, targeting could result in additional toxicity. However, we found that the HSPC population demonstrated a selective advantage and remained addicted to the forced MSI2 expression, as removal of MSI2 overexpression greatly reduced chimerism and reversed the myeloid disease. We propose that the increased expression of MSI2 in the HSPCs in high-risk MDS patients might allow for a therapeutic index in these patients. Our mouse model might provide a context to test how targeting MSI2 might alter the disease. Overall, our study suggests that targeting MSI2 could provide a therapeutic benefit in MDS.

## Methods

### Transgenic mice

*KH2-Col1A1-tet-on-MSI2/ROSA26*-rTTA transgenic mice^5^, backcrossed 10 times to C57BL/6 strain or Msi2 conditional knockout^4^ were crossed with Vav-Tg-*NUP98-HOXD13* mice. The primary donors that were used for transplants were either male or female of 3–4-month-old animals^14^. All animal procedures were approved by the Institutional Animal Care and Use Committee at Memorial Sloan Kettering Cancer Center.

### Non-competitive transplants

Transplants were performed with 2–3 × 10^6^ bone marrow cells from 12–16-week-old C57BL/6 donor mice mixed with 0.2 × 10^6^ CD45.1^+^ helper cells injected into the retro-orbital of lethally irradiated B6.SJL-Ptprc^a^ Pepc^b^/BoyJ recipient mice. Secondary transplants were performed by injecting 1 × 10^6^ bone marrow or spleen cells into sublethally irradiated B6.SJL-Ptprc^a^ Pepc^b^/BoyJ mice.

### Peripheral blood analysis

Peripheral blood was collected from the facial vein using a lancet or retro-orbital cavity using a heparinized glass capillary tube. A compete peripheral blood count was collected using a Hemavet 950 (Drew Scientific).

### Flow cytometry

Flow cytometry experiments were carried out using BD Fortessa, LSRII, or LSRFortessa instruments. Bone marrow and spleen cells collected from mice were subjected to red blood cell lysis before staining. Peripheral blood and leukaemic bone marrow and spleen were immunophenotyped with the following antibodies: CD45.2, CD45.1, Mac1, Gr1, c-Kit, CD71, Ter119 and B220. For stem cell analyses, bone marrow cells were stained with the following antibodies: lineage (Gr1, B220, CD3a, CD4, CD8 and Ter119), Sca1, c-Kit, CD150, CD48, CD16/32 and CD34. MSI2 staining was performed using a rabbit anti-mouse/human MSI2 antibody (Abcam) with a goat anti-rabbit Alexa647 conjugated secondary (Life Technologies). Anti-mouse antibodies were used at 1:200 and secondary antibodies were used at 1:400. Data analyses were performed using the FlowJo software.

### Statistical analyses

To compute *P* values for bar graphs, an unpaired 2-tailed Student's *t*-test was used except where stated otherwise. Error bars reflect the s.e.m., except where stated otherwise. In survival curves, significance was calculated using log-rank analysis. Graph Pad Prism 4.0 and the R statistical environment were used to carry out all statistical analyses.

### *NHD13* RNA-seq analysis

*NHD13* mouse RNA-seq raw data were deposited to Gene Expression Omnibus (GEO) GSE76840. Differential analysis of RNA-seq samples utilized the DESeq package for gene expression analysis^25^. False discovery rate correction of *P* values used for all bioinformatics analyses of this study utilized the Benjamini–Hochberg procedure. Mapping between entrez IDs between mouse and human genes was done using the biomaRt R packages^26^. Heatmap clustering and production was done using the heatmap function found within the NMF package in R (ref. 27).

### Human data analysis and RNA-seq analysis

The clinical microarray samples consist of 183 and 17 healthy controls from anonymized donors that were not age matched, but included elderly patients who underwent hip replacement. MDS patients and 17 controls samples were publically available on GEO with the reference series tag: GSE19429 (ref. 12). The MDS patient samples were collected from several centres: Oxford and Bournemouth (UK), Duisburg (Germany), Stockholm (Sweden) and Pavia (Italy). This study was approved by the ethics committees (Oxford C00.196, Bournemouth 9991/03/E, Duisburg 2283/03, Stockholm 410/03, Pavia 26264/2002) and informed consent was obtained^12^.

The microarray data were downloaded and had gene identifiers in the form of AffyID probes. For *MSI2* we mapped the probes and found that only 4 out of the 9 probes were correctly matched to *MSI2*. We utilized the Spearman correlation coefficient to assess the correlation with the remaining probes and the three probes, which showed a good correlation (1552364_s_at, 243010_at and 243579_at), were then averaged together. These AffyID probes were converted to human and mouse Entrez IDs, which was done using the biomaRt tool in R. The AffyIDs that had human and mouse Entrez IDs were kept as it indicated that the AffyID corresponded to a human gene that had an orthologue in mouse. In the case that several AffyIDs mapped to a single human Entrez ID the corresponding AffyID rows were combined and a mean of their values were taken. This gave one row of mean expression of all Affy probes that corresponded to a human Entrez gene.

Kaplan–Meier curves were generated to gauge survival probability between the samples that had high, low and normal *MSI2* expression. A heatmap was generated using the statistically significant genes derived from the general linearized model. The rows in this expression matrix were log10 transformed and then a *Z*-score was computed for every element in the row. A heatmap was generated using this matrix of *Z*-scores. This heatmap allowed for row and column clustering. The 200 samples which composed this matrix were labelled according to whether the sample was derived from an MDS patient or a control. Additionally, the samples were labelled according to their French–American–British MDS clinical classification. The samples could take 1 of 4 possible classifications: healthy, RA, RA with excess blasts (RAEB) and RARS. Lastly, the samples were also classified according to their *MSI2* expression levels, which could be classed as high *MSI2* expression, low *MSI2* expression or normal *MSI2* expression. *MSI2* expression was classified as high if the *Z*-score for the *MSI2* in a sample was >1. *MSI2* expression was classified as low if the *Z*-score for the musashi2 gene in a sample was <−1. *MSI2* expression was classified as normal if the *Z*-score for the musashi2 gene in a sample was −1≤ × ≤1.

### Age-matched normal individuals and primary MDS patient samples

Normal bone marrows from elderly individuals were obtained from hip replacements and MDS patient samples (PBMCs, low risk; RA *n*=3, RARS *n*=2 and high risk; RAEB-1 *n*=4, RAEB-2 *n*=6) were obtained from the Memorial Hospital Tumor Bank under the protocol IRB Waiver Number: WA0260-12 and HBUC: HBS2012060.

### Cell Cycle analysis

Before 24 h analysis, mice received an intraperitoneal injection of 1 mg kg^−1^ of BRDU. Mice were killed and Lin-Sca1+c-Kit+cells were sorted, fixed with 1.6% paraformaldehyde for 15 min, and permeablized with ice-cold methanol. To prevent cell loss, LSKs were mixed with B220+ splenocytes and subsequently stained with CD34 and Hoechst for cell cycle, and then analysed by flow cytometry.

## Additional information

**Accession codes**: *NHD13* mouse RNA-seq raw data was deposited to GEO under the accession code GSE76840.

**How to cite this article**: Taggart, J. *et al.* MSI2 is required for maintaining activated myelodysplastic syndrome stem cells. *Nat. Commun.* 7:10739 doi: 10.1038/ncomms10739 (2016).

## Supplementary Material

Supplementary Information Supplementary Figures 1-4 and Supplementary Tables 1-3

Supplementary Data 1 List of genes in the signature from the mouse NHD13/MSI2 expression program matched with human homologs.

Supplementary Data 2 Rank list of genes based on fold change comparing NHD13/MSI2 to NHD13 LSKs and used for gene set enrichment analysis.
