# Supplementary material for: MSI2 is required for maintaining activated myelodysplastic syndrome stem cells
Source: Nat Commun. 2016 Feb 22;7:10739. doi: 10.1038/ncomms10739 (PMC4764878; doi:10.1038/ncomms10739)
Supplement: Supplementary Information — Supplementary Figures 1-4 and Supplementary Tables 1-3 [file ncomms10739-s1.pdf]

Supplementary Figure 1: MSI2 is expressed in MDS and predicts poor survival.

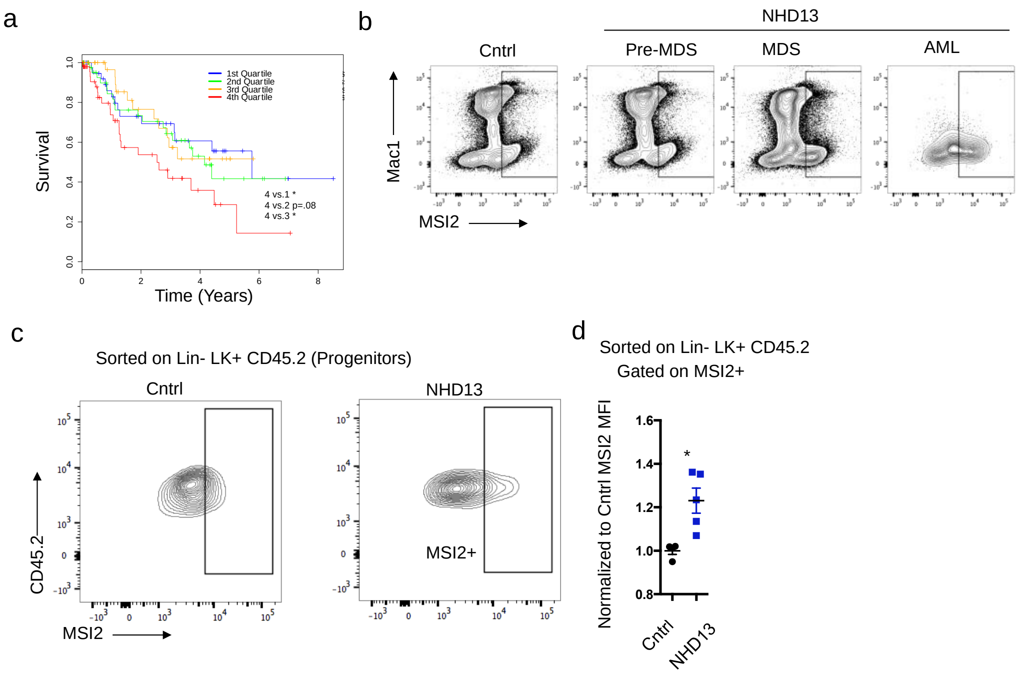

(a) Overall survival in MDS patients stratified by *MSI2* expression with quartiles (same patients as in (**Fig. 1b**). **(b)** Intracellular staining for Msi2 and Mac1 in primary transplanted animals representative gating for Figure 1e – gate indicates "Msi2 positives." **(c)** Representative gating for sorted progenitors (LK+) from control or NHD13+ transplanted mice after 3 months before MDS disease and then stained for MSI2. **(d)** Summarized flow analysis based on gating in (**c**), Control (n=4) and NHD13 (n=5) individual animals. Data in **a, b** *p<0.05 log rank and Student's *t*-test respectively **b**, horizontal line is the mean ± s.e.m.

Supplementary Figure 2: *Msi2* is required to maintain MDS.

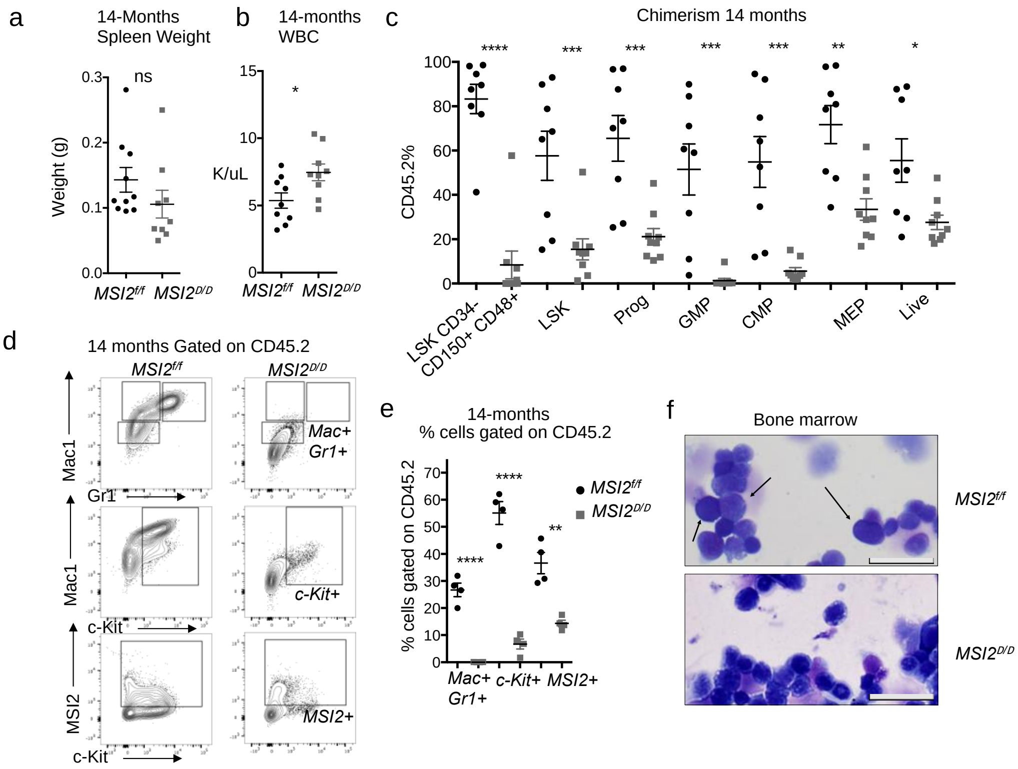

(a-f) Transplanted mice same as **Fig. 2**, mice were analyzed at 14-months post engraftment. **(a)** Spleen weights (*NHD13Msi2*$^{f/f}$ n=10*, NHD13/Msi2*$^{\Delta/\Delta}$*Mx1-Cre* n=9)**. (b)** White blood cells**. (***NHD13Msi2*$^{f/f}$ n=9, *NHD13/Msi2*$^{\Delta/\Delta}$*Mx1-Cre* n=9)**, (c)** CD45.2 chimerism (*NHD13Msi2*$^{f/f}$ n=8, *NHD13/Msi2*$^{\Delta/\Delta}$*Mx1-Cre* n=9)**. (d)** flow cytometric analysis. **(e)** Summarized flow data based on gating from **(d),** (*NHD13Msi2*$^{f/f}$ n=4, *NHD13/Msi2*$^{\Delta/\Delta}$*Mx1-Cre* n=4)**. (f)** Representative cytospins from the bone marrow and stained with Wright-Giemsa. Control mice have evidence of acute leukemia with a predominance of proerythroblasts (small black arrows) and Msi2 knockout transplanted animals have normal trilineage differentiation and no detectable blasts. White bars indicate 600x magnification with 25 m. Data in **a-c and e,** ns; not significant *p<0.05, **p<0.01, ***p<0.001, Student's *t*-test and horizontal line is the mean ± s.e.m.

Supplementary Figure 3: NHD13/MSI2 mice develop an aggressive myeloid diseases that is MSI2 dependent.

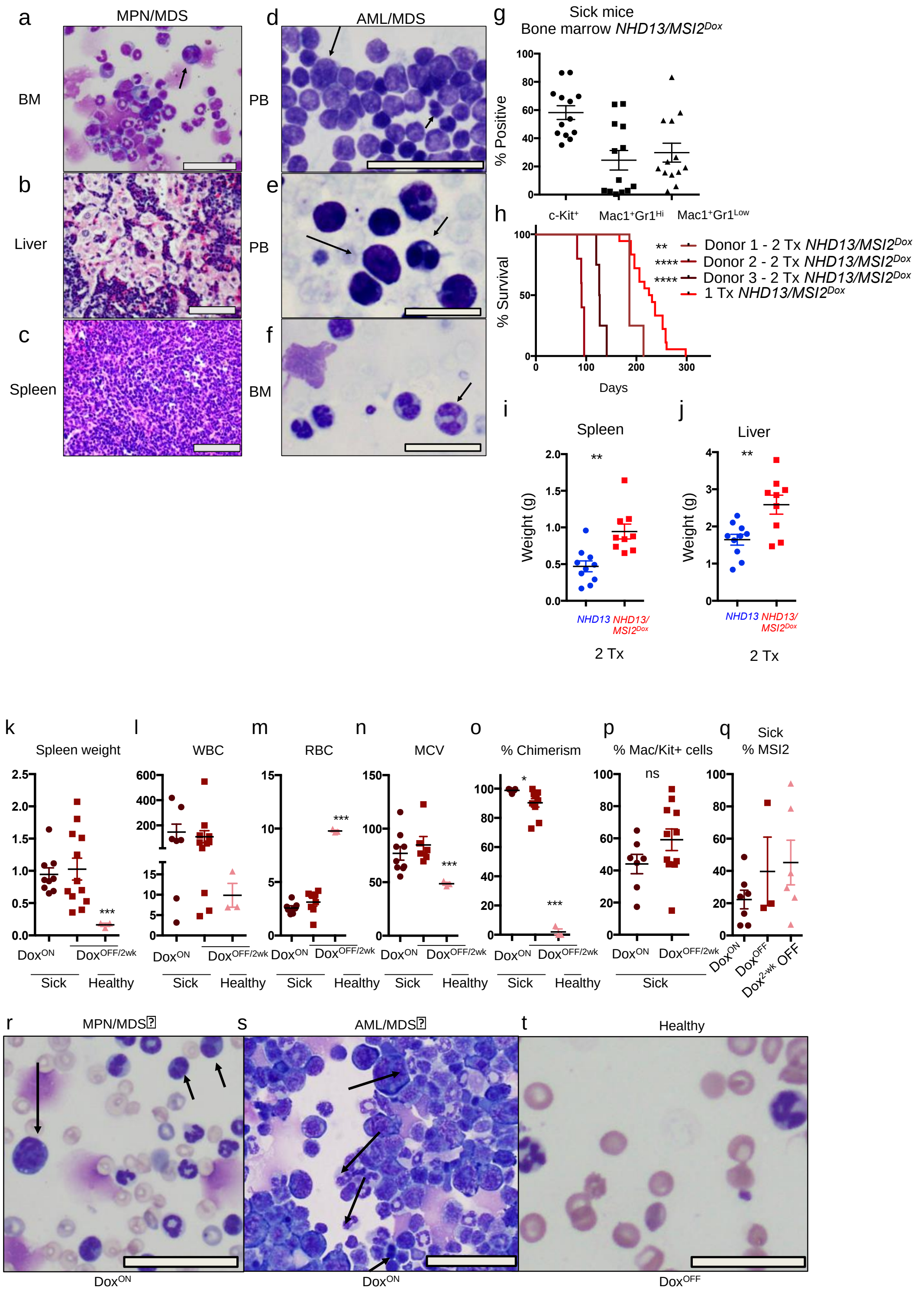

(a-f) Representative images from NHD13/MSI2^{Dox} moribound mice stained with Wright-Giemsa (peripheral blood and bone marrow cytospins) and Hematoxylin and Eosin (liver and spleen) demonstrating MPN/MDS (4 out of 12 histologically analyzed) and acute myeloid leukemia (8 out of 12). (**a**) NHD13/MSI2^{Dox} bone marrow cytospin indicating myeloid hyperplasia and dyserythropoiesis characterized by binucleate erythroid progenitors (short black arrow) from a mouse that died from an MPN/MDS. (**b-c**) Spleen and liver pathology from the same mouse in (**a**). The liver shows infiltration of the sinusoids by extramedullary hematopoiesis with dysplastic erythroid progenitors noted, and the spleen has infiltration of the red pulp by extramedullary hematopoiesis with dysplastic erythroid progenitors noted. (**d**) AML/MDS; peripheral blood smear indicates many circulating blasts (large black arrow), and dysplastic features characterized by binucleate erythroid progenitors (short black arrow), (**a-d**) White bar indicate 400x magnification with 50 μm. (**e**) Another representative mouse with AML/MDS, peripheral blood smear shows circulating blasts (black arrow), binucleate erythroid progenitors (short black arrow), and (**f**) Same mouse as in (**e**) with a hyposegmented neutrophil (pseudo-Pelger Huet neutrophil) (black arrow). White bar indicate 600x magnification with 25 μm. (**g**) Flow cytometric analysis and immunophenotype of all the mice analyzed that succumbed to hematologic disease from the NHD13/MSI2^{Dox} animals described in **Fig. 3,** (n=13 mice analyzed at endpoint). (**h**) Survival analysis of primary transplanted mice (n=18) and secondary transplant donors 1, 2 and 3 (n=4, n=4 and n=5). (**i-j**) Spleen and liver weights from secondary transplanted animals from NHD13; n=10 combined from three independent donors, NHD13/MSI2^{DOX}; n=9. (**k-s**) Data from secondary transplants that were either Dox$^{ON/OFF}$ experiments, **Fig. 3l-p**. (**k**) Spleen weights from moribund Dox^{ON} (n=9), Dox$^{OFF/OFF2wk}$ (n=12) and healthy (n=3), (l) White blood cells (WBC), moribund Dox^{ON} (n=7), Dox$^{OFF/OFF2wk}$ (n=11) and healthy mice (n=3), (**m**) red blood cell (RBC), moribound Dox^{ON} (n=7), Dox$^{OFF/OFF2wk}$ (n=8) and healthy (n=3), (**n**) mean corpuscular volume (MCV), moribund Dox^{ON} (n=9), Dox$^{OFF/OFF2wk}$ (n=6) and healthy mice (n=3), (**o**) CD45.2 donor/disease chimerism moribund Dox^{ON} (n=7), Dox$^{OFF/OFF2wk}$ (n=10) and healthy mice (n=3). (**p**) Moribund Dox^{ON} (n=7) and Dox$^{OFF/OFF2wk}$ (n=11), Mac/Kit double positive cells low or no donor chimerism in the healthy mice. (**q**) Flow cytometric analysis for MSI2 percent positivity gated on donor cells in moribund mice Dox^{ON} (n=7), Dox^{OFF} (n=3) and Dox$^{2\text{-wk OFF}}$ (n=6). (**r**) Peripheral blood smear stained with Wright-Giemsa showing MPN/MDS in Dox^{ON} mice. (**s**) Bone marrow cytospin stained with Wright-Giemsa showing AML/MDS in Dox^{ON} mice shows increased blasts dyserythropoiesis characterized by binucleate erythroid progenitors (short black arrows). (**t**) Peripheral blood smear from healthy Dox^{OFF} mouse. (r-t) White bar indicate 600x magnification with 50 μm (r) and 1000x magnification with 25 μm (s). Data in **h-j and k-q,** ns; not significant *p<0.05, **p<0.01, ***p<0.001, **h** calculated with log rank test, all other data analyzed with student's *t*-test and horizontal line is the mean ± s.e.m.

Supplementary Figure 4: Increased stem and myeloid progenitors cells in the NHD13/MSI2 mice after transplantation.

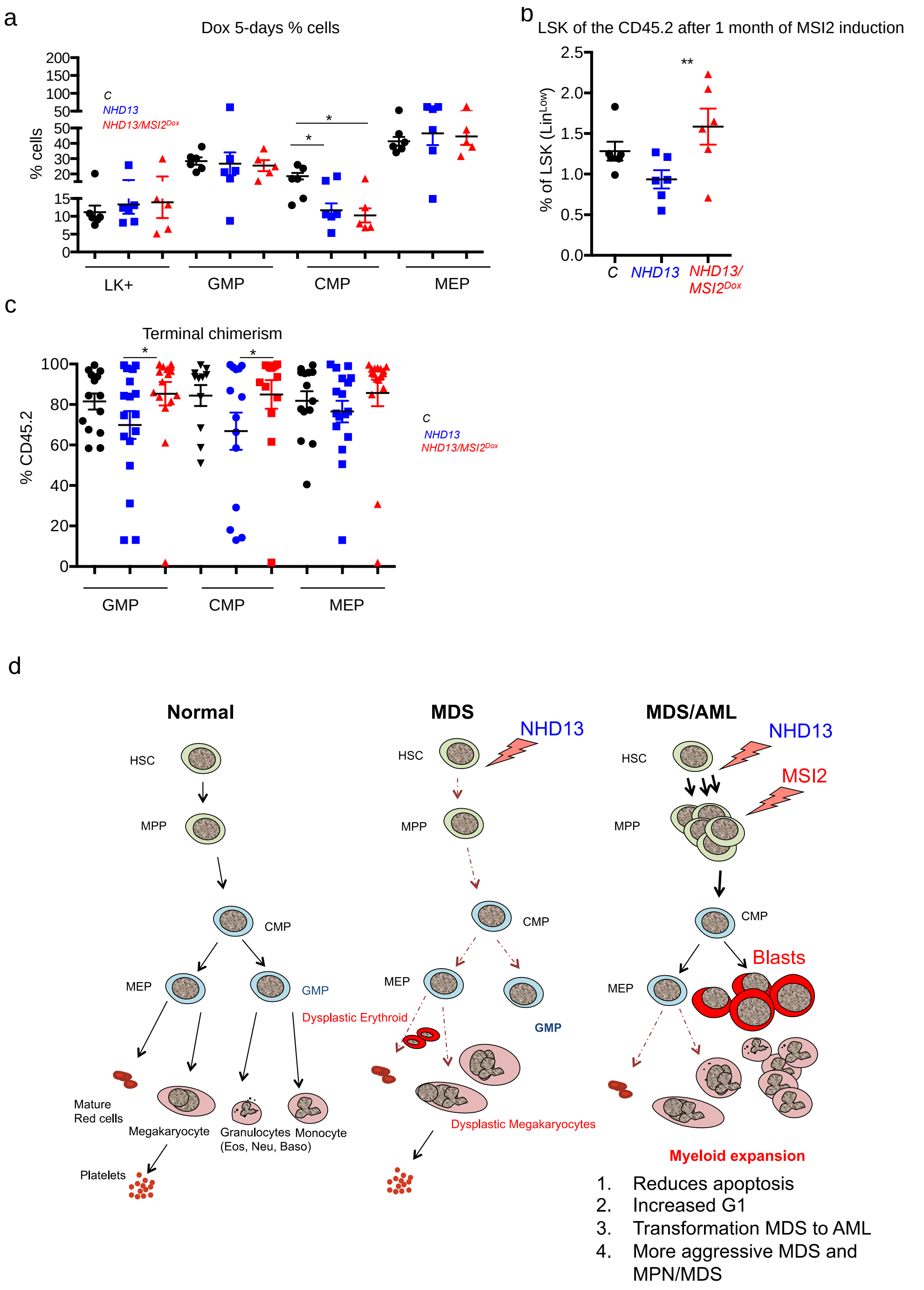

(a) Summarized flow cytometric analysis (Lineage^{low} Kit^{+}Sca1^{-}; LK+, LK+ gated; Granulocyte myeloid progenitor (GMP; CD34+,FcγRIIb+, common myeloid progenitor; CMP, CD34+,FcγRIIb^{mid}, Megakaryocyte erythroid progenitor (MEP; CD34-,FcγRIIb^{low}), primary animals treated with doxycycline (Dox) for five days and live and lin^{-} gated), (*C, NHD13, NHD13/MSI2*, n=6, n=6 and n=5 from five independent experiments) **(b)** Frequency of the LSK cell among the lineage low-gated CD45.2 cells. (C, NHD13, NHD13/MSI2^{Dox}; n=6, SEM and **p<0.01), **(c)** Terminal chimerism from transplants in Figure 3 in the gated populations, (C, NHD13, *NHD13/MSI2*^{Dox}; n=11-17 combined from two independent transplants). **(d)** In mice with NHD13 that have MDS hematopoietic development is blocked. If MSI2 is induced in the MDS mouse model, the HSPC is expanded and the mice die of an aggressive myeloid disease including leukemia. Data in **a-c** *p<0.05, **p<0.01, ***p<0.001, student's *t*-test and horizontal line is the mean ± s.e.m.

Supplementary Table 1: *MSI2* expression and correlation with mutations.

**MSI2 High expression**			**MSI2 Low expression**		
Mutation	p-value	FDR corrected	Mutation2	p-value	FDR corrected
RUNX1	0.01	0.40	TP53	0.04	1.00
SF3B1	0.05	1.00	NRAS	0.09	1.00
SRSF2	0.11	1.00	CREBBP	0.09	1.00
EZH2	0.16	1.00	CDKN2A	0.09	1.00
ASXL1	0.16	1.00	tri.19.	0.09	1.00
TET2	0.20	1.00	KDM6A	0.18	1.00
STAG2	0.20	1.00	FLT3	0.18	1.00
FLT3	0.20	1.00	rearr.3q	0.26	1.00
PHF6	0.29	1.00	abn.17	0.26	1.00
abn.17	0.29	1.00	SF3B1	0.43	1.00
tri.8.	0.44	1.00	U2AF1	0.46	1.00
U2AF1	0.50	1.00	SRSF2	0.59	1.00
DNMT3A	0.59	1.00	TET2	0.68	1.00
NRAS	1.00	1.00	ASXL1	1.00	1.00
KRAS	1.00	1.00	RUNX1	1.00	1.00
CREBBP	1.00	1.00	tri.8.	1.00	1.00
NF1	1.00	1.00	DNMT3A	1.00	1.00
WT1	1.00	1.00	EZH2	1.00	1.00
IRF1	1.00	1.00	IDH2	1.00	1.00
CDKN2A	1.00	1.00	STAG2	1.00	1.00
ETV6	1.00	1.00	ZRSR2	1.00	1.00
SH2B3	1.00	1.00	CBL	1.00	1.00
tri.19.	1.00	1.00	BCOR	1.00	1.00
TP53	1.00	1.00	JAK2	1.00	1.00
IDH2	1.00	1.00	CUX1	1.00	1.00
ZRSR2	1.00	1.00	IDH1	1.00	1.00
CBL	1.00	1.00	KRAS	1.00	1.00
BCOR	1.00	1.00	PHF6	1.00	1.00
JAK2	1.00	1.00	EP300	1.00	1.00
CUX1	1.00	1.00	GATA2	1.00	1.00
IDH1	1.00	1.00	NPM1	1.00	1.00
EP300	1.00	1.00	MLL2	1.00	1.00
GATA2	1.00	1.00	PTPN11	1.00	1.00
NPM1	1.00	1.00	KIT	1.00	1.00
MLL2	1.00	1.00	MPL	1.00	1.00
PTPN11	1.00	1.00	NF1	1.00	1.00
KIT	1.00	1.00	WT1	1.00	1.00
MPL	1.00	1.00	IRF1	1.00	1.00
RAD21	1.00	1.00	RAD21	1.00	1.00
ATRX	1.00	1.00	ATRX	1.00	1.00
KDM6A	1.00	1.00	ETV6	1.00	1.00
CEBPA	1.00	1.00	CEBPA	1.00	1.00
GS	1.00	1.00	GS	1.00	1.00
PTEN	1.00	1.00	PTEN	1.00	1.00
BRAF	1.00	1.00	SH2B3	1.00	1.00
CTN1	1.00	1.00	BRAF	1.00	1.00
rearr.3q	1.00	1.00	CTN1	1.00	1.00
del.5q.	1.00	1.00	del.5q.	1.00	1.00
del.7q.	1.00	1.00	del.7q.	1.00	1.00
del.11q.	1.00	1.00	del.11q.	1.00	1.00
del.12p.	1.00	1.00	del.12p.	1.00	1.00
del.20q.	1.00	1.00	del.20q.	1.00	1.00
del.Y.	1.00	1.00	del.Y.	1.00	1.00

Supplementary Table 2: Geneset enrichment analysis from RNA-seq positively correlated NHD13 vs. NHD13/MSI2 (upregulated).

GENESET NAME:	# of genes	ES	NES	NOM p-val	FDR q-val
CROONQUIST_NRAS_SIGNALING_UP	29	0.68	1.99	0.00	0.15
VALK_AML_CLUSTER_9	19	0.75	1.94	0.00	0.19
WANG_TUMOR_INVASIVENESS_DN	179	0.46	1.91	0.00	0.19
PID_SYNDECAN_1_PATHWAY	15	0.76	1.90	0.00	0.16
CROMER_TUMORIGENESIS_UP	17	0.73	1.89	0.00	0.15
EINAV_INTERFERON_SIGNATURE_IN_CANCER	23	0.69	1.88	0.00	0.15
ZHAN_MULTIPLE_MYELOMA_LB_DN	29	0.64	1.85	0.00	0.19
REACTOME_AMYLOIDS	31	0.60	1.85	0.00	0.18
PID_ER_NONGENOMIC_PATHWAY	27	0.65	1.82	0.00	0.22
KEGG_SYSTEMIC_LUPUS_ERYTHEMATOSUS	48	0.54	1.81	0.00	0.24
REACTOME_SIGNALING_BY_FGFR_MUTANTS	22	0.66	1.80	0.00	0.23
KIM_LRRC3B_TARGETS	14	0.74	1.80	0.00	0.22
BROWNE_HCMV_INFECTION_4HR_UP	31	0.60	1.79	0.00	0.24
REACTOME_NUCLEOTIDE_BINDING_DOMAIN_LEUCINE_RICH_REPEAT_CONTAINING_RECEPTOR_NLR_SIGNALING_PATHWAYS	39	0.57	1.78	0.00	0.24

Supplementary Table 3: Geneset enrichment analysis from RNA-seq negatively correlated NHD13 vs. NHD13/MSI2 (downregulated).

NAME	# of genes	ES	NES	NOM p-val	FDR q-val
ROSS_AML_WITH_PML_RARA_FUSION	42	-0.76	-2.06	0.00	0.00
VANHARANTA_UTERINE_FIBROID_DN	39	-0.76	-2.02	0.00	0.00
HSCVSGMP_FC3_UP	92	-0.66	-2.00	0.00	0.00
GRAHAM_NORMAL_QUIESCENT_VS_NORMAL_DIVIDING_UP	34	-0.72	-1.90	0.00	0.03
DELYS_THYROID_CANCER_DN	83	-0.63	-1.87	0.00	0.05
CHIANG_LIVER_CANCER_SUBCLASS_CTNNB1_UP	67	-0.64	-1.87	0.00	0.05
HSCVSGMP_UP	289	-0.56	-1.86	0.00	0.05
CHYLA_CBFA2T3_TARGETS_DN	121	-0.59	-1.83	0.00	0.07
ROSS_AML_OF_FAB_M7_TYPE	48	-0.66	-1.82	0.00	0.07
RAGHAVACHARI_PLATELET_SPECIFIC_GENES	52	-0.65	-1.82	0.00	0.08
HADDAD_T_LYMPHOCYTE_AND_NK_PROGENITOR_UP	43	-0.67	-1.81	0.00	0.08
SWEET_LUNG_CANCER_KRAS_DN	191	-0.55	-1.80	0.00	0.08
HSCVSMEP_FC2_DN	41	-0.67	-1.78	0.00	0.11
REACTOME_PLATELET_HOMEOSTASIS	39	-0.67	-1.78	0.00	0.11
CHIBA_RESPONSE_TO_TSA_DN	14	-0.80	-1.77	0.00	0.12
CAIRO_LIVER_DEVELOPMENT_UP	80	-0.59	-1.76	0.00	0.13
TONKS_TARGETS_OF_RUNX1_RUNX1T1_FUSION_GRANULOCYTE_UP	31	-0.70	-1.76	0.00	0.13
REACTOME_AQUAPORIN_MEDIATED_TRANSPORT	22	-0.73	-1.75	0.00	0.13
CUI_TCF21_TARGETS_DN	16	-0.78	-1.75	0.00	0.12
REACTOME_NETRIN1_SIGNALING	14	-0.81	-1.74	0.00	0.14
WENG_POR_TARGETS_LIVER_UP	22	-0.74	-1.74	0.00	0.13
GUO_HEX_TARGETS_DN	44	-0.65	-1.73	0.00	0.15
CERVERA_SDHB_TARGETS_2	38	-0.65	-1.72	0.00	0.18
VALK_AML_CLUSTER_1	15	-0.77	-1.71	0.01	0.20
KAN_RESPONSE_TO_ARSENIC_TRIOXIDE	58	-0.60	-1.70	0.01	0.22
VALK_AML_CLUSTER_8	14	-0.78	-1.69	0.00	0.23
VALK_AML_CLUSTER_2	15	-0.77	-1.69	0.01	0.23
METZELER_SURVIVALSIGNATURE_POSITIVESCORE	22	-0.69	-1.68	0.01	0.25
REACTOME_CLASS_B_2_SECRETIN_FAMILY_RECEPTORS	21	-0.71	-1.68	0.00	0.24
